# The Effect of Roasting on the Health-Promoting Components of Nuts Determined on the Basis of Fatty Acids, Polyphenol Compounds, and Antioxidant Capacity

**DOI:** 10.3390/molecules30234594

**Published:** 2025-11-29

**Authors:** Klaudia Kulik, Bożena Waszkiewicz-Robak

**Affiliations:** 1 Department of Functional and Organic Food, Institute of Human Nutrition Sciences, Warsaw University of Life Sciences, Nowoursynowska Str. 159c, 02-776 Warsaw, Poland; 2 Prof. Waclaw Dabrowski Institute of Agricultural and Food Biotechnology—State Research Institute, Rakowiecka 36, 02-532 Warsaw, Poland

**Keywords:** nuts, walnuts, hazelnuts, peanuts, convection roasting, microwave roasting, fatty acids, polyphenols, antioxidants

## Abstract

This study focused on analyzing the direction of changes in recognized health-promoting fatty acids, antioxidant activity, and total polyphenolic compound in the three most popular types of nuts, hazelnuts, walnuts, and peanuts, before and after roasting under various conditions. The roasting process caused changes in the content of selected health-promoting fatty acids in the tested nuts, which depended on both the type of nut and the roasting conditions used. The main fatty acids in walnuts are linoleic acid and α-linolenic acid, while in peanuts and hazelnuts, oleic acid was the main fatty acid. The highest losses of these acids were observed after convective roasting, and the lowest after microwave roasting with a protective coating, which promoted better preservation of these acids in the nut fat. Walnuts exhibited a relatively high antioxidant potential, which was greater than the level in peanuts and hazelnuts. Roasting (regardless of its type) increased the antioxidant potential of all tested nuts. Microwave roasting seems to be a good option in the search for optimal process conditions for the protection of health-promoting ingredients, especially since the processing time is significantly shortened.

## 1. Introduction

Tree nuts are dried fruits with a hard shell and an edible seed. They include walnuts, hazelnuts, almonds, pistachios, Brazil nuts, and peanuts. Although botanically they belong to legumes, they are classified as nuts due to their similar nutritional profile [1,2]. Due to their high nutritional value, they may have a protective effect on human health, contributing to reducing the risk of diseases such as cardiovascular diseases, type 2 diabetes, cancer, and Alzheimer’s disease [3,4].

Nuts contain large amounts of plant protein and fat-soluble active compounds (unsaturated fatty acids, phytosterols, phospholipids, phytostanols, tocotrienols, terpenoids, and squalene). They are also rich in dietary fiber, vitamins (e.g., folic acid, niacin, vitamin B6, and vitamin E), minerals (e.g., calcium, magnesium, and potassium), and numerous phytochemicals, such as phenolic acids, flavonoids, lignin, hydrolyzable tannins, proanthocyanidins, carotenoids, alkaloids, and phytates [1,5,6,7,8,9,10].

The quality of fat is extremely valuable in the context of its positive impact on human health. Nuts contain the highest percentage of fat, on average around 50%, and even up to 70–80%, making them high-energy products [11,12]. Importantly, most of the fat consists of health-beneficial unsaturated fatty acids, including essential fatty acids, which is confirmed by the results of epidemiological and clinical studies indicating that regular consumption of nuts is not a factor contributing to the development of obesity and may even help reduce body weight in the case of a low-energy diet [13,14]. Consequently, the Planetary Diet guidelines allow for a daily intake of 75 g of nuts, with the average recommended intake being approximately 50 g (twice the recommended intake of fish). At the same time, general dietary recommendations indicate an average daily intake of a minimum portion of approximately 30 g of nuts [15,16,17].

In 2003, the U.S. Food and Drug Administration approved the first health claim indicating that eating a handful of nuts daily may contribute to reducing the risk of heart disease [18]. The primary health benefits are attributed to polyunsaturated fatty acids (PUFAs) and α-linolenic acid (ALA), a precursor of bioactive n-3 fatty acids: eicosapentaenoic acid and docosahexaenoic acid. Guidelines for the prevention and dietary treatment of cardiovascular diseases state that the ratio of linoleic acid (LA) (n-6 fatty acids) to ALA (n-3 fatty acids) should be 5:1. Walnuts are particularly noteworthy among tree nuts due to their most favorable ratio of n-6 to n-3 fatty acids, which is 5.6:1 [19].

The broad health benefits of nuts also stem from the content of numerous antioxidant compounds exhibiting antiproliferative, anti-inflammatory, and anticancer properties [20,21]. Antioxidants, primarily phytosterols and polyphenolic compounds, lower the concentration of undesirable, atherogenic oxidized forms of cholesterol and reduce the risk of DNA damage. Vitamin E and monounsaturated fatty acids contained in hazelnuts stabilize the concentration of the antiatherogenic HDL cholesterol fraction, which is crucial for the prevention of type 2 diabetes [22].

Due to the fact that nutritional recommendations and research results confirming the link between nut consumption and health benefits refer primarily to their raw form, an important and interesting aspect of the research is to verify the nutritional value of their processed form, which is often chosen by consumers.

Roasting, blanching, and frying are the most common nut processing methods responsible for the development of desirable sensory characteristics, such as burnt caramel flavor and aroma, or crunchiness [23]. At the same time, the literature confirms unfavorable changes occurring during thermal processing in both the microstructure and chemical composition of nuts, including vitamins, phytosterols, phenolic compounds, and, above all, in the composition of fats and fatty acids, which may result in a deterioration of their quality and nutritional value [5,24,25,26,27,28]. Similarly, the antioxidant activity of nuts may change during roasting, but in this case, many studies confirm an enhancement of this effect [20,29].

Currently, the most popular nut processing process is roasting using convective energy, less frequently microwaves. Microwave processing, in turn, can offer many benefits (easier operation, energy savings, precise process control, faster startup and shutdown), and according to Hojjati et al. [30], this processing method can be an alternative to conventional thermal processing in the case of thawing, cooking, drying, tempering, baking, pasteurization, and sterilization [31].

Therefore, the process and type of thermal processing can determine changes both beneficial to health and potentially harmful. According to Megahed [31], roasting may result in the formation of undesirable oxidation products, such as oxysterols, epoxy and conjugated fatty acids, and fatty acid peroxides, free fatty acids, as well as compounds responsible for unpleasant taste and odor, such as guaiacol and phenylacetaldehyde.

Interestingly, roasted nuts are included in the current dietary recommendations in Malta, which is the only country among the EU countries, Iceland, Norway, Switzerland and the United Kingdom that allows the consumption of such nuts instead of fried and salted ones as part of a balanced diet [32]. According to the INC Nuts & Dried Fruits Statistical Yearbook 2024 report [33], over the past 10 years, nut consumption has been increasing, with a stable cumulative annual growth rate of 4% for highly developed countries and 7% for middle-income countries. At the same time, peanuts accounted for the majority of global peanut consumption, which grew dynamically by 2% annually. In turn, data contained in the Healthy Roasted Nut Navigating Dynamics Comprehensive Analysis and Forecasts 2025–2033 report [34] regarding the roasted nut market indicate that the market will experience dynamic growth (from $5 billion in 2025 to approximately $9 billion in 2033), with a compound annual growth rate of 5–7% between 2025 and 2033 estimated at 5–7%, driven by growing consumer awareness of health and wellness, a growing preference for convenient and nutritious snacks, and the growing popularity of plant-based diets. It is worth noting that innovations in new processing technologies, including a focus on sustainable production, are also cited as key growth factors for the processed nut market, with consumers aged 25–55, interested in health and wellness, constituting the largest segment of consumers for such products.

Therefore, the authors recognize the ongoing need for research and the search for the most optimal thermal processing method in terms of achieving both the desired technological and nutritional properties, while preserving the health-promoting nature of such an important product group as nuts.

With this in mind, and considering the need to find a processing method that meets both the sensory and nutritional expectations of consumers, the authors recognize the need to conduct research under various, reasonable conditions, as an alternative to commonly used methods.

In this study, the authors proposed an innovative microwave roasting method under precisely defined conditions, including negative pressure and vacuum. A protective coating of maltitol syrup, which is resistant to high temperatures and can provide additional protection against nutritionally beneficial compounds, was also used. The authors are aware that the raw material being tested may react differently to the roasting process depending on the variety and even the batch itself.

As a result, this study focused on analyzing the direction of changes in recognized health-promoting fatty acids, antioxidant activity, and polyphenolic compounds in the three most popular types of nuts: hazelnuts, walnuts, and peanuts, before and after roasting under various conditions.

## 2. Results

### 2.1. Fatty Acids Profile

Table 1 presents the fatty acid profile, taking into account the group of saturated fatty acids and selected mono- and polyunsaturated fatty acids identified in the fat of the tested raw nuts and those subjected to the roasting process under various conditions.

The fat composition of the examined nuts was characterized by a predominance of unsaturated fatty acids, with the highest percentage of saturated fatty acids at approximately 9% detected in microwave-roasted walnuts with a protective coating.

In the case of monounsaturated fatty acids, oleic acid was found to be the dominant fatty acid for all three types of nuts, accounting for approximately 72–82% of the fat in peanuts and hazelnuts, while in walnuts the content was approximately 7 times lower, regardless of the heat treatment used (Table 1).

Walnuts were also the type with the highest content of linoleic acid and alpha-linolenic acid, which belong to the n-6 and n-3 fatty acids considered health-promoting, respectively [35]. Compared to the other two types of nuts, walnuts had an average 9 times higher content of linoleic acid in their raw form, and after roasting, even 20 times higher than peanuts. In the case of ALA, regardless of heat treatment, walnuts contained 15%, which was about 14 times more than the other nut types studied. The roasting process caused a statistically significant change in the content of SFA and LA in the entire group of nuts studied, and these changes, like changes in the content of other fatty acids studied, depended on the type of nut studied (Table 1).

**Figure 1:**
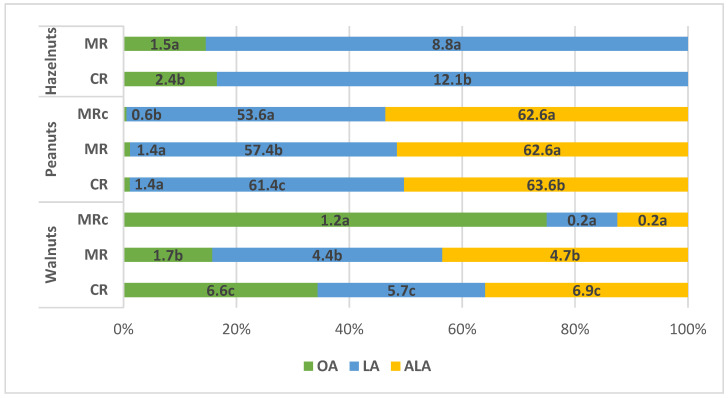
Changes in the content of main fatty acids expressed as losses after nut roasting (%). Means with different letter (a, b, c) were significantly different (*p* < 0.05).

Figure 1 shows the losses of the above-mentioned fatty acids due to roasting of nuts. Losses of LA and ALA were significantly highest after convective roasting and lowest after microwave roasting, especially for walnuts with a protective coating. Losses of oleic acid in walnuts and hazelnuts were also significantly highest after convective roasting.

**Figure 2:**
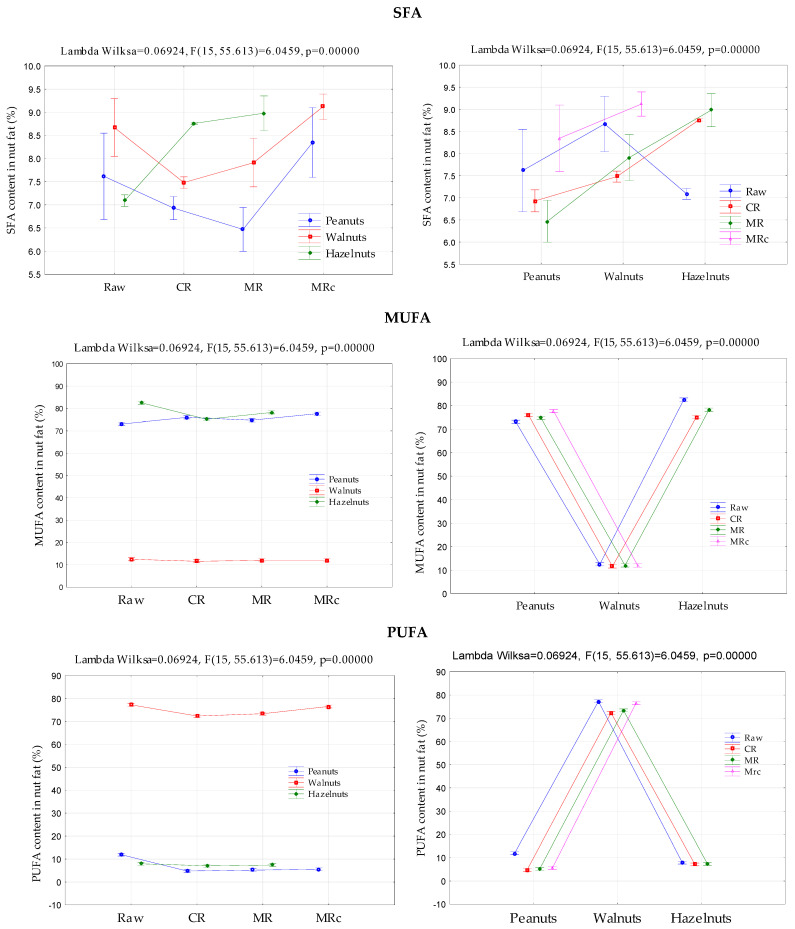
The effect of different roasting conditions and nut types on the content of fatty acids: SFA, MUFA, and PUFA. SFA—saturated fatty acids; MUFA—monounsaturated fatty acid; PUFA—polyunsaturated fatty acid. Sum of SFA: (C16:0) palmitic acid, (C17:0) margaric acid, (C18:0) stearic acid, (C20:0) arachidic acid, (C22:0) behenic acid, (C24:0) lignoceric acid; sum of MUFA: (C16:1) palmitoleic acid, (C18:1 n-9) oleic acid (OA), (C20:1) gondolanic acid, (C22:1) erucic acid; total PUFA: (C18:2) linoleic acid (LA), (C18:3) cis-9, 12,15 alpha-linolenic acid (ALA), (C20:2) cis-11,14-eicosadienoic acid, (C22:6) cis-4,7,10,13,16,19-docosahexaenoic acid (DHA).

The analysis of variance confirmed a statistically significant dependence of the content of fatty acid groups with different degrees of saturation on both the type of nuts and the roasting conditions used (*p* < 0.05) (Figure 2).

Analysis of the important parameter, the ratio of fatty acid groups, presented in Table 2, allowed us to distinguish walnuts primarily in terms of the PUFA/SFA ratio, hazelnuts and peanuts in terms of the MUFA/SFA ratio, and OA/LA fatty acids. In terms of the LA/ALA ratio, hazelnuts, especially in their raw form, were characterized by the highest value compared to other nuts.

The roasting process influenced changes in the proportions of fatty acids in nuts, while in the case of walnuts, roasting in a microwave oven, especially with the use of a protective coating, limited these changes compared to their unroasted form (Table 2).

### 2.2. Antioxidants Capacity

The total polyphenolic compound (TPC) and antioxidants properties activity against ABTS+˙ radicals and inhibition of DPPH˙ radicals in the three types of raw and roasted nuts tested are presented in Table 3.

**Figure 3:**
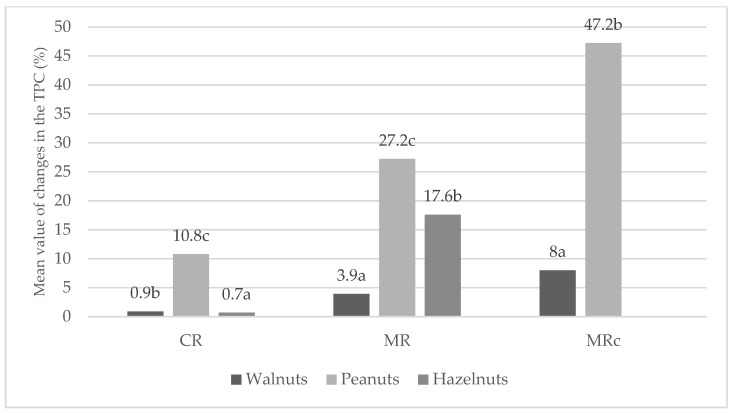
Changes in the total polyphenolic content of nuts by roasting method (%, mean value). Means with different letter (a, b, c) were significantly different (*p* < 0.05).

The highest content of total polyphenolic compounds was determined in raw walnuts (7.75 mg GAE/g), and the lowest in hazelnuts (1.36 mg GAE/g). A significant increase in polyphenolic compound content was observed in all nuts after roasting, with the highest levels observed in microwave and microwave roasting with a protective coating (Table 3 and Figure 3). Especially for peanuts, an increase in TPC during microwave roasting was observed by approximately 27% on average and almost 50% in the case of treatment both without and with a protective coating (Figure 3).

The antioxidant properties of the tested nuts were determined based on their ability to quench ABTS+˙ and inhibit DPPH radicals (Table 3).

The highest antioxidant activity against ABTS+˙ radicals was demonstrated by both raw and roasted walnuts. This activity was approximately three times greater than that of peanuts and approximately 9–10 times greater than that of hazelnuts.

Thermal treatment, such as microwave roasting, in particular, resulted in the largest statistically significant increase in this activity for each type of nut (*p* < 0.05). A similar effect was observed for the inhibition of DPPH˙ radicals (Table 3). Therefore, the radical scavenging activity of the tested nuts could be represented as follows, taking into account the roasting process used: MRc > MR > Cr > raw.

**Figure 4:**
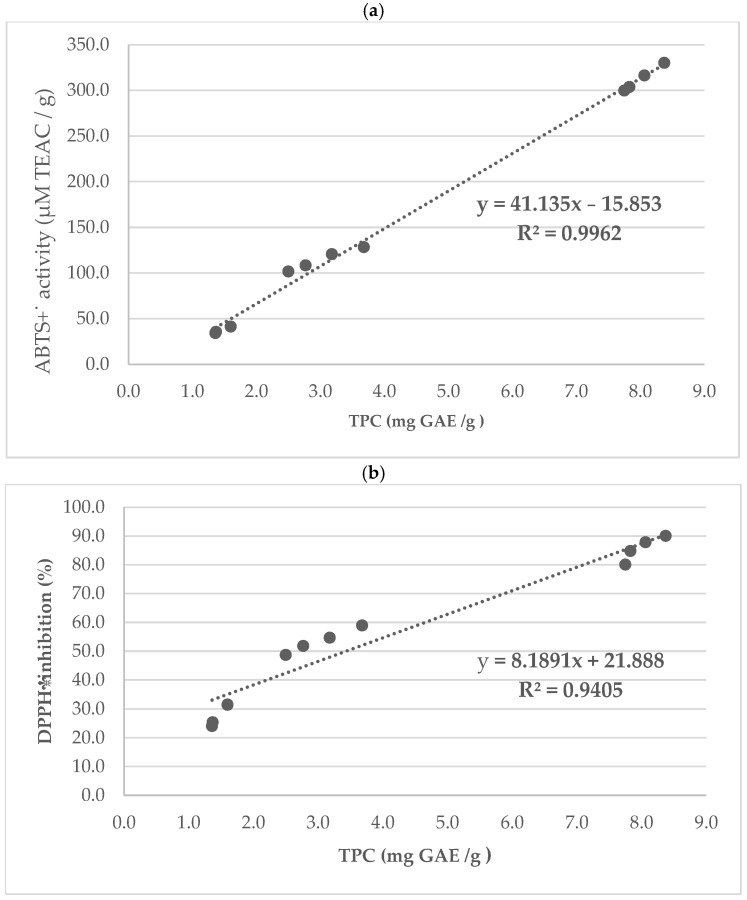
Correlation of TPC (mg GAE/g) and radical scavenging activity: (**a**) TPC and ABTS+˙ (µM TEAC/g), (**b**) TPC and DPPH˙ inhibition (%).

Figure 4 shows the correlation between radical scavenging activity and TPC in raw and roasted nuts. A good correlation was found between TPC and antioxidant activity measured against ABTS+˙ radicals (Figure 4a) and DPPH˙ radical scavenging activity (Figure 4b) (R^2^ = 0.9962 and R^2^ = 0.9405, respectively).

The higher antioxidant activity of the components in roasted nuts may result from the release or formation of new phenolic compounds.

**Figure 5:**
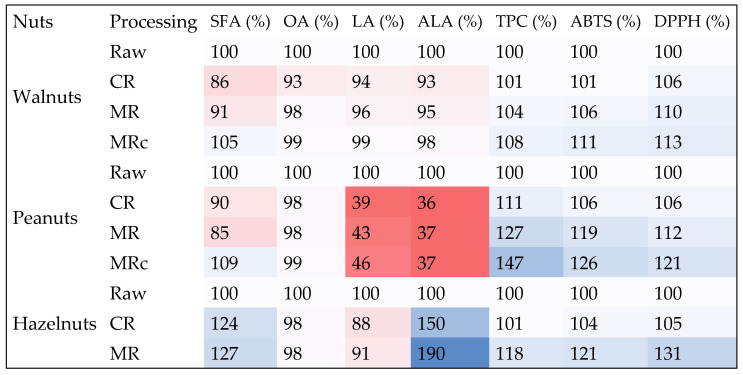
Cumulative heatmap showing the direction of changes (increase/decrease) in the tested components and indicators in nuts, taking into account their type and heat treatment (red color—significant decrease below 50%, darker/lighter pink color—decrease from approximately 2 to approximately 15%, lighter to dark blue—increase above 100%).

A heatmap is a method for visualizing the relationships, correlations, or ratios of obtained results or parameters of scientific research. To assess the impact of the nut roasting process on selected factors studied in this study, such a map was used (Figure 5), showing the level of loss or increase in components considered to be health-promoting, i.e., SFA, OA, LA, ALA, TPC, ABTS, and DPPH. The values obtained for unroasted nuts were considered the baseline value, i.e., 100%. Considering the change in color depth from red (the highest losses) through pale pink and white, and light blue, blue, and navy blue (an increase in the observed level) (Figure 5), microwave processing, both without and with a protective coating, appears to be the most beneficial with respect to the assessed changes in components, with the exception of peanuts in the case of LA and ALA.

## 3. Discussion

The health-promoting components in nuts with proven beneficial effects, including unsaturated fatty acids, minerals, protein, polyphenolic compounds, and compounds with antioxidant potential, may be subject to changes and modifications, particularly as a result of processing. The most common processing methods for nuts are blanching, frying and, above all, roasting. Therefore, the aim of the study was to determine the effect of roasting on antioxidant properties, including polyphenolic compounds, and on the fatty acid profile of nut fat, including essential fatty acids, which play a crucial role in the prevention of many diet-related diseases.

### 3.1. Fatty Acids in Nuts

Many scientific studies, including clinical trials, attribute health benefits to peanut fat, which can constitute up to 65% of the chemical composition in peanuts [36]. The SFA content is relatively low compared to the content of unsaturated fatty acids, especially MUFA, as shown in our work (from about 7% to about 9% SFA and from about 12% to about 80% OA, respectively, regardless of the type of nut and roasting method). The obtained results are comparable to the data obtained by Pedron et al. [37], Beltrán Sanahuja et al. [38] and Popovic et al. [39]. This fat composition is particularly valuable nutritionally because, as scientific sources report, it is the type of dietary fat consumed that determines plasma cholesterol levels, not its quantity alone [36]. Furthermore, a high intake of unsaturated fatty acids may help reduce the adverse effects of saturated fatty acids, while monounsaturated fatty acids lower the level of “bad” cholesterol and triacylglycerides in the blood, reducing the risk of heart attacks [36,40].

Among the nut varieties studied, hazelnuts and peanuts had the highest monounsaturated fat content, with OA being the main monounsaturated fatty acid (approximately 70–80%) in their unroasted form, which is consistent with the observations of other researchers [41,42,43]. Moreover, the literature indicates that polyunsaturated fatty acids constitute the second dominant fat fraction in hazelnuts, mainly due to the content of linoleic acid [44,45], which is also confirmed by our study. However, some studies indicate that saturated fatty acids may constitute the second major fatty acid group, which may be influenced by the higher content of saturated palmitic acid [46,47].

The fat content in walnuts is also very high, estimated at 60% on average, which depends primarily on the variety [48,49]. Walnut fat consists mainly of polyunsaturated fatty acids, while monounsaturated fatty acids are the second most important type of fatty acids, as confirmed by our own and other researchers’ results [50,51,52,53]. As expected, linoleic and linolenic acids account for the high proportion of polyunsaturated fatty acids, with oleic acid being the main monounsaturated fatty acid. OA is also the dominant fatty acid in peanuts, similarly to hazelnuts. The second group of fatty acids in peanuts are polyunsaturated fatty acids, represented by LA and ALA, although in much lower concentrations than oleic acid, as also shown in other studies [53].

Griffin et al. [54], Al-Zahrani et al. [55], and Kafkas et al. [56] indicated that each thermal treatment changes the overall fat content, especially the nutritionally valuable unsaturated fatty acids. Our study showed that the fatty acids considered dominant for a given type of nut remained unchanged, while the amounts of individual fatty acids, and thus the total content of the unsaturated and saturated fat fractions, changed.

It is desirable that the amount of polyunsaturated fatty acids in nuts does not decrease due to their positive impact on human health. Therefore, the PUFA/SFA ratio is commonly used to assess the nutritional value of oil-rich products. This ratio should be at least 0.45 [57]. As demonstrated in our study, this requirement was met for each type of nut and each roasting method (from approximately 0.45 to 9.37, Table 2). The highest value of this index was recorded for roasted walnuts (no significant difference between MR and CR roasting). The results indicate that the roasting process used in this study did not reduce the nutritional value of nut fat. However, given that unsaturated fatty acids are more susceptible to oxidation than saturated fatty acids, this presents a challenge in optimizing the storage conditions and duration of such products. The lower the value of the analyzed index, the longer the product’s shelf life. In turn, analysis of the MUFA/SFA ratio allows us to distinguish hazelnuts and peanuts in terms of this indicator. Although the observed values differed significantly by nut type and roasting method (for peanuts from approximately 6 to 6.70, for hazelnuts from approximately 7.6 to 8.6), higher values were recorded for microwave roasting for peanuts and convection roasting for hazelnuts compared to the raw form. Öz et al. [58] found, similarly to own study (Figure 2), that the type and roasting method of nuts had a significant impact on the content of SFA, MUFA, and PUFA fatty acid groups and their ratios.

It is obvious that, given the above-discussed indicators and their values, changes in the content of primarily dominant fatty acids resulting from the roasting process are significant. Such changes were noted by Alasalvar et al. [59], who determined linoleic acid content in 18 different hazelnut varieties and found a range from 3.86% to 13.77% and 4.92% to 15.70%, before and after roasting, respectively. Also, Ghazzawi and Al-Ismail [60] showed that the roasting process had a greater effect on the fatty acid composition of peanuts than the frying process (*p* < 0.05), resulting in greater losses of the important fatty acid LA (*p* < 0.05). The researchers used convective roasting at 110 °C for 16 min and frying at 175 °C for 2.5 min using refined corn oil, justifying their observations with longer processing time, which could have influenced the stronger fatty acid oxidation process. No such relationship was observed in our study; both convective and microwave roasting resulted in similar LA losses (*p* > 0.05), although the type of processing differed significantly in terms of duration (CR 15–20 min, MRc 140–180 s). In contrast, another study of walnuts showed the lowest LA content in samples roasted at 120 °C for 30 min and the highest at 150 °C for 30 min [53]. According to Kırbaşlar et al. [61], high temperatures cause strong oxidation of fatty acids, especially unsaturated ones (such as LA and ALA). In our study, in the group of nuts examined, the roasting process had a significant effect (*p* < 0.05) on changes in the content of linoleic acid and saturated fatty acids (SFA). However, in both raw and roasted nuts, oleic acid remained the dominant monounsaturated fatty acid, and linoleic acid was the dominant polyunsaturated fatty acid. Similarly, in cashew fat, Uslu and Özcan [62] observed a slight decrease from 20.20 to 18.20% in linoleic acid content after microwave roasting, while the oleic acid content slightly increased from 59.59 to 61.82%. Interestingly, cashew oil heated to 720 W contained the highest levels of linoleic and linolenic acids. Hojjati et al. [30] also confirmed that the content of unsaturated fatty acids could increase (by approximately 82%) due to microwave roasting compared to convection roasting (by approximately 74.7%) in the case of pistachios. However, with increasing microwave roasting time, a higher percentage of saturated fatty acids was observed compared to linoleic and oleic acids.

It was shown that not every roasting method used significantly reduced the content of monounsaturated and polyunsaturated fatty acids. Statistical analysis of our results revealed that microwave processing conducted at 60 °C and pressure of 40 hPa was a stronger factor protecting their oxidation, particularly microwave roasting with a protective coating.

The share of OA, ALA, and LA is extremely important nutritionally, because essential unsaturated fatty acids, including LA and ALA, can be converted and then metabolized into pro-inflammatory eicosanoids [63].

Popovic et al. [39] observed no significant changes in the fatty acid profiles of walnuts after 12 h of heating at 60 °C, although the content of unsaturated fatty acids was higher after roasting than the initial value. Moreover, the authors defined the average LA/ALA ratio was 5.76 in raw walnuts and 5.94 in roasted walnuts, which was higher than the values obtained in our study for this type of nut. Nevertheless, considering that the favorable these fatty acid ratio ranged from 1:1 to 4:1, the results obtained for walnuts and peanuts, regardless of the roasting method, are very favorable (Table 2).

Another important parameter of processed fat quality assessed is the oleic acid to linoleic acid (OA/LA) ratio, which is an indicator of resistance to oxidative damage during refining and storage of oils. High values of this parameter are technologically desirable because they determine a long shelf life [64].

Uquiche et al. [65] noticed that microwave food processing can also generate desirable changes such as increasing the oxidative stability of Chilean hazelnut oil, and when used as a pre-treatment process for obtaining cold-pressed peanut oil, it results in a better taste and extends its shelf life [66].

In our own studies, we observed a higher OA/LA ratio for hazelnuts (all processed) and peanuts (microwaved), which can be explained by the higher OA content in these nuts compared to walnuts. As observed by Kirbaslar and Erkmen [67], oxidation of linoleic acid occurs during hazelnut roasting, but the authors state that the process conditions are crucial. Applying a temperature of 135 °C for 20 min results in a higher degree of oxidation than applying a temperature of 120 °C for a longer time, i.e., 30 min, which in turn results in an increase in the content of unsaturated fatty acids (OA and LA).

O’Keefe et al. [68] obtained different observations, showing no significant effect of the roasting process on the total oleic acid content in hazelnut fat (*p* > 0.05). This was hypothesized to be due to the fact that monounsaturated fatty acids (MUFA) are more resistant to oxidation than polyunsaturated fatty acids (PUFA) during thermal roasting. In this context, they reported fatty acid oxidation rates of 10:100:200 for OA, LA, and ALA, respectively.

It is worth noting that in our study, no significant increase in the total SFA content of walnut and peanut samples was observed after microwave and convection roasting. It should also be noted that the results obtained in our study indicate that roasted nuts can still be a source of health-promoting fatty acids.

### 3.2. Antioxidant Capacity

According to the literature, the antioxidant potential and high total phenolic content of nuts also determine their beneficial health-promoting effects. It is this antioxidant potential that is credited with protecting important nutrients, such as health-promoting fatty acids, tocopherols, and minerals, from degradation [69,70].

It is widely known that polyphenols are a large group of key plant phenolic compounds, including flavanoids, phenolic acids, polyphenolamides, lignans, stilbenes, tannins, and curcuminoids, which possess antioxidant and anti-inflammatory activity [71,72]. The level of phenolic compounds in nuts, as well as the above-described composition and content of fat and fatty acids, depend on many factors, such as environmental factors, soil composition, degree of ripeness, but also food processing, including thermal treatment [73].

The most commonly used method for determining total polyphenolic compounds is the Folin–Ciocalteu colorimetric method, which was used in this study. The nut varieties studied differed significantly in their raw TPC content, with walnuts having the highest TPC content compared to the other nuts, approximately 7 times more than hazelnuts and approximately 3 times more than peanuts (Table 3). Hazelnuts, on the other hand, had the lowest TPC content compared to the other nuts, but convective roasting did not change their level content (*p* < 0.05).

According to literature data, the conditions of convective roasting, especially for hazelnuts, are within the temperature range of 100–160 °C for 10–60 min, which corresponds to the conditions used in our own research [74]. Ciemniewska-Żytkiewicz et al., 2015 [75] observed that the total phenolic content in roasted hazelnuts increases, and their higher share is observed in nuts roasted at 160 °C than in the range of 100–130 °C. The values obtained as a result of thermal treatment by the researchers are relatively similar to the values obtained in our own research, assuming that we compare the ratio of changes, as the obtained initial TPC value in hazelnuts is approximately two times lower.

In turn, the TPC content in peanuts observed in our study was higher than that determined by Win et al. [76], Fakhriya et al. [77] and Ghazzawi and Al-Ismail [78]. In our results, the TPC content in raw peanuts was the same as that observed in roasted peanuts at temperatures ranging from 170 to 180 °C for 2.5 to 3 min using refined corn oil as the heating medium by Ghazzawi and Al-Ismail [78]. The researchers also found that convective roasting at 110–120 °C for 15–17 min significantly increased TPC (by approximately 4%) compared to raw peanuts. Comparing our results, TPC for convectively roasted peanuts increased (by approximately 10%), while microwave treatment resulted in an increase of approximately 27%. According to the literature, the increase in antioxidant activity and oil content in microwave-roasted peanuts may also be due to significant water loss. The convective roasting conditions at 160 °C for 10 min used by Win et al. [76] can be compared to our microwave treatment, as a similar level of TPC increase was observed in peanuts as in our study. Roasting of MR with a protective coating resulted in an increase in TPC of up to approximately 47% (Table 3).

Oliveira et al. [42] and Salve et al. [79] also noted that thermal processing of nuts, including roasting, increases TPC in almonds and peanuts, justifying the observations with the release of phenolic substances or phenolic analogs after the destruction of cellular structures in roasted kernels, which may react with the Folin–Ciocalteu reagent [80,81]. Sobolev [81] also pointed out that during pressure cooking, some peanut components, such as lignans, hydrolyze to form vanillin, which combines with TPC, increasing their total amount. Such transformations may be enhanced in walnuts, as they are considered one of the main plant sources of polyphenolic compounds [82].

Compared to the two types of nuts tested, it was observed that walnuts were stronger antioxidants, which is consistent with the observations of other researchers [83] and may be due to the simultaneous presence of other components, including tocopherols [84].

Pereira et al. [48] demonstrated that the walnut variety also generates variations in TPC content. They showed that the total phenolic content of six different walnut varieties grown in Portugal ranged widely from 58.8 to 95.1 mg GAE/g, while Arranz et al. [80] estimated the average total phenolic content of the same walnut variety at 10.7 mg GAE/g, which was significantly higher than our observations.

Wangcharoen and Gomolmanee [85] observed a linear relationship between TPC and flavonoid content and antioxidant activity in boiled peanuts (100 °C for 60 min). In our study, a good correlation was also observed between TPC and DPPH˙ inhibition (R2 = 0.94) and anti-ABTS+˙ activity (R2 = 0.99) (Figure 4).

The DPPH˙ radical is commonly used to assess antioxidant activity in vitro, and the method is based on measuring the reducing capacity of antioxidants towards the DPPH˙ radical [86]. Literature data on the effect of heat treatment on the antioxidant activity of nuts, measured by DPPH˙ radical scavenging, are inconclusive.

In our study, roasting resulted in an increase in the antioxidant activity of nuts (Table 3). The highest values were found for walnuts (*p* < 0.05), while in the case of peanuts they were about two times lower, and in the case of hazelnuts they were about 3–3.5 times lower than in walnuts.

According to Açar et al. [86], heat treatment can significantly alter DPPH˙ radical activity. For example, in pine nuts and almonds, an increase was observed following roasting, while frying reduced it. Salve et al. [79] also confirmed that DPPH˙ radical scavenging activity was significantly higher in processed peanuts compared to raw peanuts, with the highest levels observed in fried and pressure-cooked peanuts, followed by roasted peanuts. However, they also noted that ABTS+˙ radical scavenging activity did not differ significantly between raw and roasted peanuts, which is consistent with our results for raw and convectively roasted hazelnuts and peanuts. In turn, Popovic et al. [39] observed an increase in antioxidant activity (*p* < 0.05) for roasted walnuts, both for DPPH˙ radicals and for ABTS+˙ radicals.

Differences in the observed increases or decreases in antioxidant activity against ABTS+˙ or DPPH˙ radicals may result from the mechanism of action of the antioxidant compounds. According to Gulcin’s study [87], antioxidant activity can operate via a hydrogen atom transfer or single electron transfer reaction. Therefore, nuts may have a different reaction mechanism after heat treatment or a combination of both mechanisms, which may react antagonistically or synergistically, and thus may lead to variations in the level of scavenging activity.

Elouafy et al. [88] and Li et al. [53] confirmed, in line with our observations, that walnuts have a very high antioxidant capacity, and roasting has a direct effect on antioxidant activity by increasing the power and ability of the fat to scavenge DPPH˙ and ABTS+˙ free radicals (*p* < 0.05). Roasting the nuts at 120 °C for 20 min resulted in a significant increase in ABTS+˙ values, and the highest DPPH˙ value was recorded at 120 °C for 10 min. The roasting process may enhance the antioxidant properties of walnut fat, and the significant content of tocopherols (vitamin E) may act as an essential antioxidant, stimulating protection against degradation and loss of these properties [89]. At the same time, Tunasamy et al. [90] observed otherwise, not confirming the increased antioxidant activity of roasted walnuts against ABTS+˙ radicals compared to their raw form.

In the case of almonds, Oliveira et al. [91] confirmed that the mean activity against DPPH˙ and ABTS+˙ radicals increased after roasting, and positive correlations were observed between total phenolic content and ABTS+˙ and between total phenolic content and DPPH˙, similar to our results (Table 3, Figure 4). Such correlations have been previously reported by Oliveira et al. [92] and in studies on walnuts [48].

According to Açar and colleagues [86], roasting nuts can destroy some bioactive compounds, but it may also be responsible for the formation of compounds with antioxidant activity. As explained by Salin-Sanchez [93], the increase in total phenolic content and antioxidant activity may be due to (1) the degradation of cellular components during roasting and the release of previously retained phenolic acids into the cellular environment, (2) the varying antioxidant activity of phenolic compounds during intermediate oxidation stages, and (3) the induction of new compounds with high antioxidant activity, associated with thermal processing. Maillard reaction products, particularly melanoidins, may contribute to the total phenolic content and antioxidant activity [26]. Therefore, the amount of phenolics and antioxidants may vary depending on the heat treatment used [94]. It is worth noting that phenolics are one of the important indicators of nut quality. According to Francisco and Resurreccion [95] it is the group of these compounds together with flavonoids that is responsible for the color, characteristic taste and aroma after roasting.

The balance between thermal degradation of natural antioxidants and the formation of new compounds with antioxidant activity is also largely dependent on the roasting process. Schlörmann et al. [11] observed that roasting can lead to a reduction in the antioxidant activity of some nuts (hazelnuts and walnuts), but in others (almonds and pistachios), the activity remains stable or is increased. This reduction in activity is due to the loss of polyphenols due to heat treatment, but the formation of compounds with antioxidant activity can offset this effect, as indicated by Król et al. [96] for roasted hazelnuts.

## 4. Materials and Methods

### 4.1. Materials

The following samples were selected for the study: walnuts (*Juglans regia* L.)—country of origin: USA, Chandler variety; hazelnuts (*Corylus avellana*)—country of origin: Azerbaijan, Ata-Baba variety; and shelled peanuts (*Arachis hypogaea* L.)—country of origin: Nicaragua—Runner variety. The samples came from Bakalland S.A., Łódź, Poland.

The moisture content of raw, roasted and unroasted nuts was tested using the AOAC dryer method [97]. The moisture content of the nuts was stable throughout the storage period and averaged: 4.25% for raw walnuts, 6.08% for peanuts, and 5.09% for hazelnuts. After roasting, the moisture content of all conventionally roasted nuts ranged from 2.52 to 2.96%; for microwave-roasted nuts, from 0.44 to 0.99%; and for microwave-roasted nuts with a protective coating, from 0.47 to 0.99%.

To ensure consistency and comparability of results, a 5 g sample was used in each replicate. To ensure consistency and comparability of results, all nut samples were stored under controlled conditions prior to analysis.

Immediately after roasting, the nuts were cooled to ambient temperature (20 °C), then sealed in barrier packaging in a protective atmosphere (N_2_) and stored in controlled conditions at 4 °C without access to light until chemical analyses were performed. The storage period did not exceed 1 week, and all samples were analyzed within the same time frame.

### 4.2. Roasting Conditions

Three types of roasting were used:

(1) Convection (CR)—convective roasting was carried out using a laboratory rotary oven (Petrocini, Sant Agostino, Italy). Dry roasting was carried out at 170 °C (i.e., the temperature used during industrial roasting of nuts at the Bakalland factory, Warsaw, Poland). Due to the diverse structure of nuts, the most favorable individual roasting time for each type was selected by trial-and-error (by visual inspection), which was 15 min for walnuts and peanuts and 20 min for hazelnuts.
(2) Microwave—laboratory-scale (MR), roasting was conducted under negative pressure in a microwave-vacuum dryer (PROMIS-TECH Sp. z o.o., Wrocław, Poland), roasting pressure 40 hPa, temperature 60 °C, time varying for individual nuts (due to their different structure and size): 140 s for walnuts and peanuts and 180 s for hazelnuts.
(3) Microwave with a protective coating (MRc)—roasting was conducted under the same conditions as for microwave roasting without a protective coating (as described above). The device was additionally equipped with a system for automatic, uniform spraying of sugar syrup onto walnuts and peanuts, as determined by the company from which the samples were obtained. Industrial maltitol syrup (83°Bx) was used, which, due to its very high viscosity, was diluted with water at a 1:1 (m/m) ratio.

### 4.3. Methods

All chemical analyses were performed in at least three replicates for at least two technological trials.

#### 4.3.1. The Fatty Acid Composition

The fatty acid composition was determined in accordance with PN–ISO5509:2001 [98] and PN–EN–ISO 5508:1996 [99] standards. Separation and identification of fatty acids were performed in a gas chromatograph coupled to a Clarus SQ 8T mass spectrometer (Perkin Elmer, Waltham, MA, USA) equipped with an Elite-WAX capillary column (length 30.0 m, internal diameter 0.25 mm, film thickness 0.25 μm). The injection temperature was 250 °C. The initial gas chromatograph oven temperature was 60 °C. After 2 min, the temperature increased at a rate of 6 °C/min to 250 °C and maintained under these conditions for 10 min. The carrier gas (helium) flow in the column was 1.10 mL/min. A 1:20.0 flow splitter (Perkin Elmer, Waltham, MA, USA) was used. The ion source and junction temperatures were 220 °C and 220 °C, respectively. The mass spectrometer was operated in total ion current mode. The mass range scanned was 50–450 amu. Fatty acids were identified by comparing their mass spectra and retention times with standards (NCP-GLC-674, Nu-Chek-Prep Inc., Elysian, MN, USA).

##### Calculation of Changes in the Content of Selected Fatty Acids Expressed as Losses After Nut Roasting (%)

The loss of selected fatty acids after roasting the nuts was calculated according to the following formula:

FA loss % = (*A* − *B*)/*A* × 100,

in which:

FA—fatty acid/s
*A*—FA content before roasting (raw nuts);
*B*—SFA content after roasting (CR, MR, MRc).

#### 4.3.2. Extraction of Phenolic Compounds and Antioxidants

Phenolic compounds of nuts were extracted according to Slatnar Et Al. [100] with some modification.

Nuts, regardless of the type, were ground in a coffee grinder (BOSCH TSM6A013B, BSH Sprzet Gospodarstwa Domowego Sp. z o.o., Warsaw, Poland) for 3 min, and then 1 g of sample were added to 10 mL of mixture of methanol: water (80:20, *v*/*v*). The mixture sonicated in water-bath for 30 min, followed by centrifugation at 5000 rpm for 15 min. The supernatant was filtered through a nylon filter. The analysis was conducted in triplicate.

##### Total Polyphenolic Content (TPC)

Total polyphenolic compound content was determined by the Folin–Ciocalteu colorimetric method. This method utilizes the color-reactive properties of polyphenols with the Folin–Ciocalteau reagent. The Folin-Ciocalteau reagent (0.5 mL) (Sigma Aldrich, Merck Darmstadt, Germany) was added to the methanolic extract of the sample (10 mL). Then, after adding 2 mL of sodium carbonate solution, the tube contents were mixed thoroughly (2 min) and the final volume was made up to 25 mL with distilled water. Following the addition of 2 mL of sodium carbonate solution tubes were mixed and the final volume was completed to 25 mL with distilled water. At the end of incubation time (1 h, room temperature), total phenolic content was determined—the absorbance measured at 750 nm in a spectrophotometer (Shimadzu UV-Vıs spectrophotometer, UV mini 1240, Shimadzu, Kyoto, Japan). The results of quantitative determinations were calculated by reference to the absorbance for the standard substance (gallic acid) based on the calibration curve and expressed as the number of gallic acid equivalents, mg GAE (Gallic Acid Equivalent) per 100 g of nuts [mg GAE/100 g] [101]).

##### Calculation of TPC After Roasting

The losses of TPC after roasting the nuts was calculated according to the following formula:

TPC loss % = (*A* − *B*)/*A* × 100,

in which:

*A*—TPC content before roasting (raw nuts);
*B*—nut TPC content after roasting (CR, MR, MRc)

#### 4.3.3. Antioxidant Properties

Antioxidant properties were determined using two research methods:

(1) The method of Brand-Williams et al. [102], modified by Thaipong [103]. This method involves the reduction in the stable azo radical DPPH (1,1-diphenyl-2-picrylhydrazyl) by antioxidants contained in the sample. Color was measured spectrophotometrically at a wavelength of λ = 517 nm (Shimadzu UV-2401 spectrophotometer, Kyoto, Japan, for both assays). Results were expressed as % DPPH radical inhibition.
  % Inhibition = ((Absorbance sample (nut) − Absorbance control))/(Absorbance control) × 100
(2) Antioxidants reduce ABTS+˙ (oxidized form) to colorless ABTS (reduced form). The decrease in absorbance is a measure of the antioxidant content in the tested material. The measurement was taken at a wavelength of 734 nm. Antioxidant content is expressed as Trolox equivalents (after taking into account the conversions resulting from the standard curve) as μM TEAC (Trolox Equivalent Antioxidant Capacity), calculated per 1g of the tested material.

#### 4.3.4. Statistical Methods

Statistical analysis was performed using Microsoft Office Excel 2007 and Statistica 13.3.

The obtained results were described using parameters such as arithmetic mean and standard deviation (SD). The normality of data distribution was verified using the Shapiro–Wilk test (α = 0.05), and the homogeneity of variance was assessed using Levene’s test (α = 0.01). Duncan’s test (α = 0.05) was applied to examine differences between individual groups.

Statistical differences were determined using analysis of variance (factors were nut type and roasting method). No significant differences (*p* ≥ 0.05) were designated as non-significant (NS).

#### 4.3.5. A Heatmap as a Way to Visualize the Impact of the Roasting Process on Selected Components Important for Human Health

A heatmap is a data visualization method that uses colors to represent data values, making it easier to spot positive and negative changes, such as the loss of or increase in components relevant to the study. Warmer colors, such as orange or red, may indicate a lower content or decrease in the level of a given component, while cooler colors, such as white, blue, or navy blue, indicate a higher level or an increase in the level of the component being assessed.

The cumulative heatmap presented in this paper shows the direction of change as a percentage change (increase/decrease) in the tested components and indicators in nuts, taking into account their type and heat treatment (red color—significant decrease below 50%, darker/lighter pink color—decrease from approximately 2 to approximately 15%, lighter to dark blue—increase above 100%). All determined values of components such as: SFA (%), OA (%), LA (%), ALA (%), TPC (%), ABTS (%), DPPH (%) for raw nuts were considered as the initial value, i.e., 100%.

## 5. Conclusions

Nuts are a good source of many compounds with recognized health-promoting properties, such as fat, including essential fatty acids, and polyphenolic compounds.

This study demonstrated that heat treatment, so important for the organoleptic benefits obtained, can result in both positive and negative changes to the nutritional value of nuts.

The use of the roasting process caused changes in the content of selected health-promoting fatty acids in the tested nuts, which depended on both the type of nut and the roasting conditions used.

The main fatty acids in walnuts were LA and ALA, while in peanuts and hazelnuts, OA was the most abundant. The highest losses of these acids were observed after convective roasting, and the lowest after microwave roasting with a protective coating, which promoted the better preservation of these acids in the nut fat.

Walnuts exhibited a relatively high antioxidant potential, which, with respect to ABTS+˙ radicals, was approximately three times greater than in peanuts and approximately 10 times greater than in hazelnuts. The antioxidant potential of walnuts was also higher than that of hazelnuts and peanuts against DPPH˙ radicals.

In summary, heat treatment has a significant impact on the health-promoting properties of nuts, causing changes that can be controlled by selecting appropriate conditions, monitoring the process, and conducting research on the quality of the finished product, especially in the context of protecting fat, which largely determines the product’s suitability for consumption.

Considering the results of our own work, microwave roasting seems to be a good approach in the search for optimal process conditions for protecting health-promoting components, especially since the processing time is significantly reduced. At the same time, the authors see the need to continue and supplement such research with results regarding other components, both present, emerging, and undergoing transformation, particularly those with antinutritional properties.

## Figures and Tables

**Table 1:** Effect of roasting type on fatty acids profile in raw nuts (%, mean value ± SD).

Nuts	Raw	CR	MR	MRc	Significance	
Saturated fatty acids (SFA) (%)					Nuts	Roasting
Walnuts (*n* = 9)	8.67 ± 0.25 b	7.48 ± 0.05 a	7.91 ± 0.21 a	9.12 ± 0.11 b		
Peanuts (*n* = 9)	7.62 ± 0.38 b	6.93 ± 0.10 a	6.47 ± 0.19 a	8.30 ± 0.37 c	*	*
Hazelnuts (*n* = 9)	7.09 ± 0.05 a	8.76 ± 0.01 b	8.98 ± 0.15 b	n/s		
Oleic acid C18:1 (OA) (%)						
Walnuts (*n* = 9)	12.15 ± 0.67 d	11.35 ± 0.50 a	11.94 ± 0.34 b	12.00 ± 0.09 c	*	
Peanuts (*n* = 9)	73.43 ± 0.10 b	72.43 ± 0.10 a	72.37 ± 0.14 a	73.00 ± 0.26 b		
Hazelnuts (*n* = 9)	81.95 ± 0.15 c	80.02 ± 0.10 a	80.73 ± 0.18 b	n/s		
Linoleic acid C18:2 (LA) (%)						
Walnuts (*n* = 9)	61.58 ± 0.86 c	58.09 ± 0.86 a	58.86 ± 0.72 b	61.48 ± 0.43 c		
Peanuts (*n* = 9)	8.56 ± 1.03 d	3.30 ± 0.10 a	3.65 ± 0.48 b	3.97 ± 0.31 c	*	*
Hazelnuts (*n* = 9)	7.92 ± 0.22 b	6.96 ± 0.10 a	7.22 ± 0.02 b	n/s		
Alpha-linolenic acid C18:3 (ALA) (%)						
Walnuts (*n* = 9)	15.18 ± 0.09 c	14.14 ± 0.13 a	14.46 ± 0.33 b	14.94 ± 0.41 bc		
Peanuts (*n* = 9)	2.97 ± 0.11 c	1.08 ± 0.05 a	1.11 ± 0.09 b	1.11 ± 0.03 b	*	
Hazelnuts (*n* = 9)	0.10 ± 0.03 a	0.15 ± 0.07 b	0.19 ± 0.10 b	n/s		

CR—convectional roasting; MR—microwave roasting; MRc—microwave roasting with a protective coating. Means with different letter (a, b, c, d) in the same row were significantly different (*p* < 0.05); n/s—non studied. *—significance *p* < 0.05.

**Table 2:** Summary and comparison of the ratios of groups and selected fatty acids important for human health (%, mean value).

Nuts	Raw	CR	MR	MRc
PUFA/SFA *				
Walnuts (*n* = 9)	8.75 a **	9.76 b	9.37 b	8.21 a
Peanuts (*n* = 9)	0.95 c	0.40 a	0.46 b	0.44 b
Hazelnuts (*n* = 9)	0.76 b	0.82 c	0.72 a	n/s
MUFA/SFA				
Walnuts (*n* = 9)	1.41 b	1.56 c	1.53 c	1.28 a
Peanuts (*n* = 9)	6.19 b	6.32 c	6.74 c	5.99 a
Hazelnuts (*n* = 9)	7.93 b	8.61 c	7.63 a	n/s
OA/LA				
Walnuts *(n* = 9)	0.19 a	0.19 a	0.20 ab	0.19 a
Peanuts (*n* = 9)	8.57 a	22.16 d	20.01 c	18.43 b
Hazelnuts (*n* = 9)	10.35 a	10.74 b	10.77 b	n/s
LA/ALA				
Walnuts (*n* = 9)	4.05 a	4.10 b	4.07 a	4.14 b
Peanuts (*n* = 9)	2.88 a	3.06 b	3.29 c	3.61 c
Hazelnuts (*n* = 9)	79.2 c	46.10 b	38.01 a	n/s

* PUFA—polyunsaturated fatty acid; SFA—saturated fatty acids; MUFA—monounsaturated fatty acid. Sum of SFA: (C16:0) palmitic acid, (C17:0) margaric acid, (C18:0) stearic acid, (C20:0) arachidic acid, (C22:0) behenic acid, (C24:0) lignoceric acid; sum of MUFA: (C16:1) palmitoleic acid, (C18:1 n-9) oleic acid (OA), (C20:1) gondolanic acid, (C22:1) erucic acid; total PUFA: (C18:2) linoleic acid (LA), (C18:3) cis-9, 12,15 alpha-linolenic acid (ALA), (C20:2) cis-11,14-eicosadienoic acid, (C22:6) cis-4,7,10,13,16,19-docosahexaenoic acid (DHA). ** Means with different letter (a, b, c, d) in the same row were significantly different (*p* < 0.05). n/s—non studied.

**Table 3:** Effect of roasting on the TPC and antioxidant activity of raw nuts (mean value ± SD).

Nuts	Raw	CR	MR	MRc
Total phenolic content (TPC) (mg GAE/g)				
Walnuts (*n* = 9)	7.75 ± 0.25 a *	7.82 ± 0.04 b	8.05 ± 0.01 c	8.37 ± 0.04 d
Peanuts (*n* = 9)	2.50 ± 0.15 a	2.77 ± 0.07 b	3.18 ± 0.08 c	3.68 ± 0.05 d
Hazelnuts (*n* = 9)	1.36 ± 0.05 a	1.37 ± 0.02 a	1.60 ± 0.15 b	n/s
ABTS+˙ activity (µM TEAC/g)				
Walnuts *(n* = 9)	299.69 ± 1.78 a	303.76± 0.48 b	316.32 ± 1.37 c	330.17 ± 1.97 d
Peanuts (*n* = 9)	101.59 ± 0.93 a	108.11 ± 0.57 b	120.66 ± 0.84 c	128.33 ± 0.83 d
Hazelnuts (*n* = 9)	34.02 ± 0.60 a	35.53 ± 0.90 a	41.22 ± 0.93 b	n/s
DPPH˙ inhibition (%)				
Walnuts (*n* = 9)	80.08 ± 0.24 a	84.82 ± 0.34 b	87.83 ± 0.25 c	90.6 ± 0.41 d
Peanuts (*n* = 9)	48.67 ± 1.18 a	51.82 ± 0.47 b	54.68 ± 0.28 c	58.93 ± 0.60 d
Hazelnuts (*n* = 9)	24.07 ± 0.24 a	25.37 ± 1.30 b	31.42 ± 1.23 c	n/s

* Means with different letter (a, b, c, d) were significantly different (*p* < 0.05). n/s—non studied.

## Data Availability

The original contributions presented in this study are included in the article. Further inquiries can be directed to the corresponding author.

## Abbreviations

The following abbreviations are used in this manuscript:

CR	Convectional roasting
MR	Microwave roasting
MRc	Microwave roasting with a protective coating
FA	Fatty acid/s
SFA	Saturated fatty acid/s
PUFA	Polyunsaturated fatty acid/s
ALA	Alpha-linolenic acid C18:3
LA	Linoleic acid C18:2
OA	Oleic acid C18:1
TPC	Total polyphenolic compound
ABTS+˙	2,2′-azinobis(3-ethylbenzothiazoline-6-sulfonic acid
DPPH˙	2,2′-azinobis(3-ethylbenzothiazoline-6-sulfonic acid
